# DNA repair-dependent immunogenic liabilities in colorectal cancer: opportunities from errors

**DOI:** 10.1038/s41416-024-02848-8

**Published:** 2024-09-13

**Authors:** V. Amodio, P. P. Vitiello, A. Bardelli, G. Germano

**Affiliations:** 1 IFOM ETS - The AIRC Institute of Molecular Oncology, 20139 Milan, Italy; 2 Department of Oncology, Molecular Biotechnology Center, University of Torino, 10126 Turin, Italy; 3 Department of Medical Biotechnologies and Translational Medicine, University of Milano, 20133 Milan, Italy

**Keywords:** Oncology, Colorectal cancer

## Abstract

Colorectal cancer (CRC) remains one of the major causes of cancer death worldwide. Chemotherapy continues to serve as the primary treatment modality, while immunotherapy is largely ineffective for the majority of CRC patients. Seminal discoveries have emphasized that modifying DNA damage response (DDR) mechanisms confers both cell-autonomous and immune-related vulnerabilities across various cancers. In CRC, approximately 15% of tumours exhibit alterations in the mismatch repair (MMR) machinery, resulting in a high number of neoantigens and the activation of the type I interferon response. These factors, in conjunction with immune checkpoint blockades, collectively stimulate anticancer immunity. Furthermore, although less frequently, somatic alterations in the homologous recombination (HR) pathway are observed in CRC; these defects lead to genome instability and telomere alterations, supporting the use of poly (ADP-ribose) polymerase (PARP) inhibitors in HR-deficient CRC patients. Additionally, other DDR inhibitors, such as Ataxia Telangiectasia and Rad3-related protein (ATR) inhibitors, have shown some efficacy both in preclinical models and in the clinical setting, irrespective of MMR proficiency. The aim of this review is to elucidate how preexisting or induced vulnerabilities in DNA repair pathways represent an opportunity to increase tumour sensitivity to immune-based therapies in CRC.

## Introduction

DNA repair mechanisms are central to suppressing tumour onset by restricting the emergence of mutations due to physiological DNA replication and/or caused by endogenous and exogenous sources of DNA damage [1]. The inactivation of DNA repair mechanisms is associated with cancer initiation and progression in many types of cancer [1, 2]. A subset of colorectal cancers (CRCs) is characterized by germline and/or somatic genetic defects in DNA damage response (DDR) genes. The prevalence of somatic DDR defects in colorectal cancer (CRC) ranges between 10 and 30% [3–5] and the prevalence of DDR gene alterations is affected by the side of the colon where the disease emerges. There is evidence that the histology and molecular and immunological landscapes of CRCs arising on the right or left side of the colon are distinct [6].

**Fig. 1:**
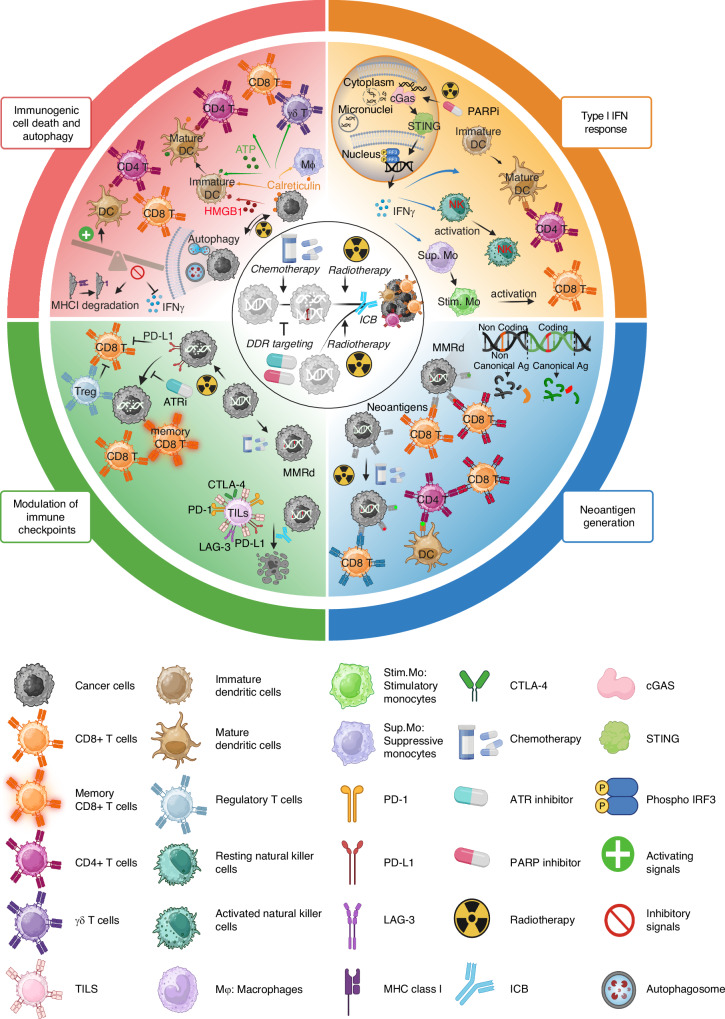
Defective DNA repair pathways can reshape the immune microenvironment of CRCs. Radiotherapy, chemotherapy, and targeted agents against DNA repair pathways can potentially engage the host’s immune system when combined with immune modulator molecules. Impairing the DDR leads to micronuclei formation and cytosolic DNA fostering cGAS-STING activation and consequently releasing cytokines that are part of the type I IFN response (orange panel). This, in turn, promotes NK activation, immune activating monocyte polarization and dendritic cell maturation, facilitating the presentation of MHC class II neoantigens to CD4 + T cells (orange panel). DNA mutations are a source of canonical and non-canonical neoantigens in Mismatch Repair deficient tumours (blue panel). Chemotherapy and radiotherapy can lead to promote the neoantigen levels in cancer (blue panel). Moreover, chemotherapy treated or mismatch repair deficient tumours show high expression of immune checkpoints such as CTLA-4, PD-1, PD-L1 and LAG3 on tumour infiltrating lymphocytes (green panel). Additionally, the radiation-mediated upregulation of PD-L1 on cancer cells is impaired by ATR inhibitors, which dramatically decrease the number of tumour-infiltrating Tregs (green panel). Altered DDR pathways may be involved in the immunogenic cell death programme through the release of HMGB1, ATP and calreticulin exposure on the cell surface. This engagement activates innate and adaptive immune cells, enhancing anti-tumoural immune control (red panel). Additionally, DDR alterations induce autophagy, augmenting antigen presentation and increasing the survival function while regulating the homoeostasis of CD8 + T cells (red panel). Conversely, autophagy can inhibit type I IFN response and degrades MHC class I leading to immune escape (red panel). Sup. Mono suppressive monocytes, Stim. Mono stimulatory monocytes, Mφ macrophages, Ag antigen.

In CRC, mismatch repair (MMR) was the first DNA repair pathway to be linked to prognosis and response to therapy [7]. Notably, genetic alterations and epigenetic silencing of genes belonging to the MMR machinery are frequent in CRCs, with 15% of cancers in stages I-III being MMR-deficient (MMRd). These tumours are associated with right-sided primary tumours, older age at diagnosis and female sex [8]. In the clinic, MMRd tumours exhibit high response rates to immune checkpoint blockade (ICB). For example, pembrolizumab, an anti-programmed death 1 (PD-1) agent, has shown remarkable efficacy in MMRd patients in whom two or more lines of treatment have failed [9]. In addition, pathogenic variants of other DDR pathways, such as homologous recombination (HR), are also frequent in CRC [5]. HR is a DNA repair process that provides high-fidelity, template-dependent repair or tolerance of complex DNA damage, including DNA gaps, DNA double-strand breaks (DSBs), stalled replication forks and DNA interstrand crosslinks [10]. The contribution of HR alterations in CRC was highlighted by the association of germline pathogenic variants of the breast cancer gene 1 (*BRCA1*), a key player in the HR machinery, with an increased risk of CRC in a meta-analysis [11]. Unlike MMRd, HR deficiency (HRd) is not associated primarily with the side of the primary tumour, and recent studies have elucidated how HRd can be exploited to treat CRC [5, 12, 13]. As observed in ovarian and breast tumours, targeting poly (ADP-ribose) polymerase 1 (PARP1) in tumours with *BRCA1* and breast cancer gene 2 (*BRCA2*) inactivation selectively causes cancer cell death, showing promise in clinical settings [14, 15]. This mechanism, known as synthetic lethality, is due to the simultaneous loss of function of two genes. In this context, the pharmacological blockade of PARP1 generates DNA single-strand breaks (SSBs), whose persistence results in DSBs that cannot be properly repaired when HR is inactive [5]. In this review, we address how alterations in DNA repair affect the immune system of the host (Fig. 1). Several findings suggest that the high number of neoantigens in MMRd tumours is crucial for the immune response [16, 17]. In addition, micronuclei (nucleus-like structures) arise from fragmented chromosomes upon DNA damage. The chromosomes contained in the micronuclei acquire DSBs and, following the disruption of the micronuclei envelope, trigger the molecular activation of intracellular pathways, such as the cyclic GMP-AMP synthase-stimulator of interferon gene (cGAS-STING) pathway, which is also linked to immune surveillance (Fig. 1, orange panel) [18, 19]. Whether and to what extent these concepts can be translated from MMR to other DDR pathways have been the focus of recent studies and are addressed in the following sections. Moreover, ongoing clinical strategies aimed at increasing immunogenicity and sensitivity to ICB by inducing DNA damage via chemotherapy/radiotherapy will also be presented.

In summary, this review highlights mechanistic, translational, and clinical studies describing how the DDR can be targeted in CRC in combination with ICB to trigger antitumour immunity.

### The unique biological and clinical features of MMR-deficient CRC

Mismatch repair is a multicomplex protein system that is required for the detection and replacement of single-nucleotide mismatches, in addition to large and small deletions that escape proofreading during replication [20]. Both genetic and epigenetic alterations in MMR genes occur early in colorectal carcinogenesis, influencing subsequent genetic events [21]. For this reason, CRCs are classified into two subgroups: (a) MMR proficient (MMRp) tumours, which maintain a constant length of microsatellites and are thus defined as microsatellite stable tumours (MSS), in which chromosomal instability is the main driver of genomic instability, and (b) MMRd tumours, in which the length of the microsatellite regions changes frequently during cell division, inducing microsatellite instability (MSI) [20]. At the genomic level, MMRd tumours accumulate many frameshifts (FSs) and single-nucleotide variants (SNVs) and are characterized by a high mutational burden [20, 22]. The majority of MMRd/MSI CRCs are the result of somatic mutations in MMR genes or the epigenetic downregulation of MutL Homolog1 (*MLH1)* expression [23].

As many as 28% of MMRd/MSI CRCs occur within the context of hereditary nonpolyposis colorectal cancer (HNPCC), also known as Lynch syndrome (LS) [24]. LS is a hereditary cancer syndrome characterized by heterozygous germline mutations in *MLH1*, MutS protein homologue 2 (*MSH2)*, MutS protein homologue 6 (*MSH6)* or PMS1 Homologue 2 (*PMS2)* [24]. In addition, a small fraction of patients develop MSI-high tumours due to biallelic mismatch repair deficiency (BMMRD) syndrome, which is associated with the very early onset of gliomas and colorectal and endometrial tumours [25]. The relative abundance of MMRd tumours decreases from 15% (stages I-III) to 5% in advanced (metastatic) settings, establishing MMRd status as a positive prognostic factor in local disease [26–28]. Notably, the involvement of the immune system was not systematically considered until a decade ago, despite the long-known abundance of lymphocyte infiltration in MMRd/MSI CRC [29–31]. The initial causative association between MMRd status and immunogenicity stems from a series of observations that were rightfully connected: the finding that somatic mutations are substantially increased by 10-100-fold in MMRd tumours compared with MMRp tumours [32, 33] and the realization that somatic mutations found in tumours can be recognized by the patient’s own immune system [34]. These observations were contextualized when examining the complete response in a single patient with metastatic CRC, in a molecularly unselected population, during an early phase clinical trial with an anti-PD-1 agent [35], paving the way for the first successful clinical trial assessing anti-PD-1 treatment for MMRd tumours [9]. Several investigations have mechanistically associated the inactivation of the MMR system with the accumulation of neoantigens, which are the drivers of the immune response upon immunotherapy [17, 36]. We recently reported that MMRd immunogenicity relies not only on canonical neoantigens (derived from the coding part of the genome) but also on noncanonical neoantigens (derived from introns, 5ʹ and 3ʹ UTRs, or alternative readings of the coding regions), which represent the majority of the total neoantigens generated by MMR inactivation (Fig. 1, blue panel) [37]. Furthermore, the role of dendritic cell activity in expanding the diversity of CD8 + T cell responses and harnessing neoantigen content for therapeutic benefit in MMRd tumours has recently been explored [38].

In parallel, while it is widely accepted that the high number of neoantigens in MMRd tumours represents the main trigger for initiating an immune reaction, other mechanisms that contribute to effective antitumour immunity have been proposed. Recently, the impact of c-GAS-STING pathway activation following the release of cytosolic DNA has been highlighted as central to generating a robust immune reaction in MMRd cancers by activating the type I interferon (IFN-I) response (Fig. 1, orange panel) [19, 39, 40]. Gajewski and colleagues elegantly described the role of cGAS-STING pathway activation in cancer, emphasizing how cancer outcomes are influenced by the successful priming of T cells by activated antigen-presenting cells in the tumour microenvironment. In particular, they showed that the activation of pathways involving dendritic cells (DCs) that lead to the production of IFN-I is pivotal for antigen-mediated priming of CD8 + T cells [41]. Additionally, cGAS-STING activation enhances natural killer (NK) cell activation, cytotoxicity, and antitumour effects in many tumour models independently of CD8 + T cells (Fig. 1, orange panel) [42]. Kwart and colleagues reported that cGAS-STING activation and the IFN-I response can transform immunosuppressive monocytes into immunostimulatory monocytes [43], whereas another group reported that IFN-I attenuates regulatory T cell function [44]. How a favourable microenvironment in MSI-high tumours affects the positive outcomes of these tumour types was examined by Llosa and colleagues [45]. The differential expression of immune checkpoint proteins between MMRd and MMRp tumours has been demonstrated. Cytotoxic T-lymphocyte antigen 4 (CTLA-4), PD-1 and its ligand (PD-L1), and lymphocyte-activation gene 3 (LAG3) are expressed at higher levels in tumour-infiltrating lymphocytes, in the stroma and in the invasive front of MMRd tumours than in MMRp tumours (Fig. 1, green panel) [45]. This effect is likely a consequence of the immune-responsive tumour microenvironment due to the high neoantigen levels of these tumour types rather than the direct upregulation of immune checkpoint expression in MMRd cancers [45]. Interestingly, the biology of MMRd tumours differs from that governing other immunogenic tumour types. Indeed, we and others have shown that MMRd CRCs can respond to ICB despite frequent alterations in antigen-presenting genes. Notably, we found that patients whose tumours had impaired beta 2 microglobulin (B2M) expression responded to ICB treatment, even in the absence of antigen-presenting machinery, and the same results were reported in mice lacking the CD8 + T cell compartment [46]. Consistent with these findings, the Voest’s laboratory recently demonstrated that MSI-high tumours with loss of B2M still respond to ICB, and that this effect was associated with an elevated frequency of activated γδ T cells in ICB-naïve tumours and an increased presence of γδ T cells in the tumour microenvironment after ICB treatment [47].

This wealth of biological information on the mechanisms of MMRd immunogenicity has been paralleled by the clinical development of ICB in the management of early [48, 49] and advanced CRC [50–52], leading to the approval of anti-PD-1 +/- anti-CTLA-4 blocking antibodies in the treatment of MMRd/MSI-H CRCs. In addition, in 2017 the Food and Drug Administration (FDA) approved pembrolizumab (an anti-PD-1 agent) for the treatment of MSI or MMRd tumours regardless of histology or anatomical location, making this the first tissue-agnostic indication in oncology [53].

The relationship between the genetic features and response to immunotherapy of CRC tumours has been confirmed in multiple prospective trials. In the metastatic setting, approximately 45% of MSI CRCs present a tumour objective response upon first-line ICB, with a duration of response that exceeds 36 months in more than 75% of patients [51]. These results indicate that ICB treatment has great therapeutic success in a small subgroup of MMRd metastatic CRCs (<5% of the total), although primary and acquired resistance restrict the efficacy of the treatment, as thoroughly discussed elsewhere [54–56].

### The majority of CRC patients do not benefit from ICB therapies

As discussed, ICB is ineffective in MMRp CRC patients, who account for the vast majority (85–95%) of cases [9, 57, 58]. The evidence of the insensitivity of CRC patients to ICB emerged in a pivotal study by Le, where the immune-related objective response rate and 20-week progression-free survival rate were 0% and 11% respectively, for patients with MMRp CRC [9]. The immunological background behind the unresponsiveness of the majority of MMRp CRC patients to ICB is characterized by a paucity of immune cells [45]. Furthermore, seminal observations from the Jack’s group highlighted how MSS CRC patients contain clonal neoantigens. However, these neoantigens are broadly expressed at lower levels than in MMR-deficient cancer patients, thus affecting productive cross-priming and driving T cell dysfunction [59]. Despite this limitation, the authors demonstrated that the administration of anti-CD40 improves the efficacy of anti-PD-1 and anti-CTLA-4 in terms of primary tumour and metastasis formation in CRC without MMR deficiency [59]. The authors hypothesized that agonist antibody against CD40 receptor enhances the costimulatory function of antigen-presenting cells by generating new T cell responses against weak affinity or poorly expressed neoantigens [59].

However, some exceptions are emerging regarding the immunogenicity of MMRp CRCs [58, 60, 61]. In the NICHE trial (ClinicalTrials.gov: NCT03026140), Chalabi and colleagues reported that up to 27% of early (stage I-III) MMRp CRCs exhibited a pathological response to the combination of nivolumab and ipilimumab given as neoadjuvant treatment [60]. With respect to metastatic MMRp CRC, recent evidence supports the efficacy of new ICB agents in treating this typically immune refractory disease at the metastatic stage. In particular, the combination of balstilimab (an anti-PD-1 antibody) with botensilimab (a novel Fc-enhanced anti-CTLA-4 antibody) has shown an overall response rate (ORR) of 22% in a population of patients with non-liver metastases (NLMs), who were preferentially enroled in the expansion phase of the trial [61]. Fc-enhanced anti-CTLA-4, in addition to blocking CTLA-4 ligand interactions, has an increased affinity for FcγRs on antigen presenting cells (APCs) and NK cells [61]. This promotes T cell priming, activation, and memory formation through improved coengagement of APCs and T cells [62]. Interestingly, the potential activity of ICB agents in NLM MSS metastatic CRC (mCRC) patients has already been reported in another early phase trial, based on a different ICB regimen [63]. The same detrimental effect of liver metastases in response to ICB has been noted in patients with MSI CRCs treated with ICB as a first-line therapy [64]. However, a molecular explanation regarding the difference in response to immunotherapy in patients with and without liver metastases is not yet available but may be related to the different features of the tumour microenvironment (TME), as already shown for non small cell lung cancer (NSCLC) [65]. Regarding the relationship between cancer-specific molecular drivers and immunogenicity, experimental evidence is accumulating regarding the impact of genetic dependencies in the beta-catenin, RAS/BRAF or PI3K/PTEN pathways on the composition of the TME [66, 67]. However, we still lack the clinical correlation needed to incorporate such information as potential biomarkers in the management of MMRp or MMRd CRCs.

### Beyond the dichotomy between MMRp and MMRd tumours

Different genetic or epigenetic degrees of deficiency of the MMR system are emerging as relevant factors in determining the response of MMRd tumours to ICB treatment [16, 68]. Interestingly, clinical results in glioma patients with constitutive BMMRD, which characterize the most hypermutant tumours (typically 100-1000 mutations per Mb), suggest that ICB treatment can be effective by retreatment with ICB in combination with other agents even after tumour progression [69]. These results suggest that different types and degrees of MMR inactivation are correlated with distinct immunogenic levels.

Although cancers can be classified as MMRp or MMRd, recent findings point to the presence of heterogeneous (functional or nonfunctional) status of the MMR machinery within the same tumour. In fact, evidence suggests that cells, expressing or not MMR proteins, can coexist in the same tumour [70–73]. While in some cases atypical MMR immunohistochemical staining is due to technical artefacts, tumours containing cells with different molecular backgrounds and MMR statuses indeed exist as observed by several authors; this has multiple biological, clinical, and therapeutic implications [71, 74, 75]. In 2014, Joost and colleagues analysed areas of a CRC with heterogeneous MLH1 protein expression, and reported that areas lacking MLH1 expression presented *Mlh1* promoter methylation and a shift in microsatellite length [76]. The heterogeneous loss of MMR protein expression can be classified as intraglandular (within or between glandular formations), clonal (in whole glands or groups of glands) or compartmental (in larger tumour areas/compartments or between different tumour blocks) [76]. We recently reported that the coexistence of MMRp and MMRd cells in the same tumour niche affects immune infiltration and immune surveillance in a preclinical model of CRC [77]. Our results suggest that the immune response against the MMRd component could also be effective towards the MMRp counterpart and indicate that CD8+ and γδ T cells are involved in antitumoural immune response. However, whether this is the result of a bystander effect or by other mechanisms requires further investigation [77]. Interestingly, albeit limited to a few cases, clinical data are available on the impact of MMR heterogeneity on the immune response in CRC. For example, Loupakis and colleagues described a case in which a patient harbouring a metastatic CRC tumour characterized by MMR heterogeneity and treated with nivolumab and ipilimumab experienced prolonged disease control [75].

The loss of expression of MMR proteins can have different layers of complexity. Berrino and colleagues reported that spatial heterogeneity of one of the four main MMR proteins (MLH1, MSH2, MSH6, or PMS2) could be identified in the context of the complete loss of expression of other components of the MMR system [78]. This observation led to the identification of an MMR heterogeneous pattern characterized by areas of double loss of expression of MMR proteins in MMRd tumours. Importantly, the authors reported that “double loss MMR” tumours harboured increased levels of exhausted CD8 + T cells, suggesting that these tumours may be less responsive to ICB [78].

MMR heterogeneity can also be observed at the intraindividual level, resulting in a discordant MMR status between the primary tumour and secondary lesions [79, 80], but it can also occur at the intertumour level in patients with synchronous or metachronous multiple primary CRCs [81]. This evidence suggests that ideally, in the presence of multiple lesions (metastases or multiple primary tumours), an assessment of the MMR status of each tumour site would be informative of the tumour biology and possibly pivotal in designing a personalized therapeutic approach for each patient.

On the other hand, intratumour MMR heterogeneity is relatively infrequent [74, 75], although the number of MMR heterogeneous CRCs described could be underestimated for several reasons. First, pathological investigations are usually performed on a single tumour block or on endoscopy biopsies and may therefore not adequately represent the complexity of the tumour mass [74]. Second, variability in pathological evaluation across different institutions could underestimate the extent of this phenomenon [74]. Accordingly, efforts to improve liquid biopsy procedures for monitoring the presence of tumour components with different MMR status in the blood might be beneficial in clinical practice, especially in cases characterized by genetic alterations in MMR genes. In conclusion, while the existence of MMR heterogeneity is recognized, its impact on tumour immune surveillance and the response to immunotherapy remains to be thoroughly dissected.

### Altered DNA double-strand repair triggers an immune-fertile microenvironment in CRC

Double-strand breaks resulting from exogenous and endogenous DNA damage can be repaired through nonhomologous end joining (NHEJ) or HR mechanisms [1]. NHEJ, is a rapid and high-capacity pathway that joins two DNA ends with minimal sequence homology, whereas HR requires extensive homology through a sister chromatid template [82]. The utilization of these repair pathways is dependent on the cell cycle phase and the nature of the DNA ends [83]. The classical NHEJ (cNHEJ) pathway is initiated by the Ku70-Ku80 heterodimer, which binds the DNA ends and recruits DNA-dependent protein kinases (DNA-PKcs). Additional proteins, such as the nuclease Artemis and DNA polymerases Polμ or Polλ are involved in the generation of compatible ends, which are finally ligated by the complex including DNA ligase IV and other factors [82, 84]. Alternatively, DSBs can be resected, resulting in 3’ single-stranded DNA available for HR, single-strand annealing (SSA), and alternative end-joining (alt-EJ) repair mechanisms. HR is initiated by the MRN (MRE11, RAD50 and NBS1) complex, c-terminal binding protein (CTBP) interacting protein (CTip) and other exonucleases, whose activity generates 3’-single-stranded DNA. This acts as a substrate for coating and stabilization, which is mediated by replication protein A (RPA). BRCA2 promotes the replacement of RPA with RAD51, a nucleoprotein that catalyses strand invasion and permits the extension of the DNA end via the intact sequence on the sister chromatid as a template. SSA is a long homology-directed repair that results in the loss of genetic material and involves RAD52 and DNA ligase I [84].

Classical NHEJ is the major DSB repair pathway in the G0/G1 cell cycle phases but also functions in the S/G2 phases. HR is active in S/G2 cell cycle phases where sister chromatids are in proximity, providing a homologous template. Except DSBs produced by replication fork collapse, which are almost exclusively repaired by HR [85], the competition between cNHEJ and HR for repairing DSBs in S/G2 phases is managed by CYREN, a cell cycle-dependent inhibitor of cNHEJ [86].

Alt-EJ is a microhomology direct repair mechanism in which PARP1, X-ray repair cross-complementing protein 1 (XRCC1) and DNA ligase III lead to the insertion and deletion of large fragments affecting DNA fidelity. NHEJ is considered error-prone due to potential sequence gain or loss, whereas HR is defined as error free, utilizing a sister chromatid template. However, this is considered an oversimplification considering that the quality of DSBs (DSBs with complementary overhangs for NHEJ and perfect homology for HR) affects the quality of the repair independently of the DNA damage response pathway involved [85].

Importantly, NHEJ plays a critical role in repairing naturally occurring DSBs, as observed in variable diversity-joining recombination. During this process, DSBs are intentionally created to generate antigen receptor genes of early B and T cells and to enable class switching [87]. These pathways are frequently mutated in cancer, and alterations via chromosomal aberrations and/or genetic and epigenetic inactivation of the aforementioned proteins lead to HRd in multiple tumours [88]. Like MMRd, HRd is characterized by distinct genomic features such as loss of heterozygosity, telomeric allelic imbalance, and large-scale state transitions [5]. These features, together with the presence of inactivating mutations in the *BRCA1* and *BRCA2* genes, are captured by genomic diagnostic tests currently used in clinical practice to identify HRd ovarian and breast tumours that are more likely to benefit from PARP1 inhibitors [5]. Notably, a single heterozygous mutation in HR genes does not translate to HRd status, and only biallelic alterations result in the acquisition of the genomic features of ineffective HR [89]. This is of particular interest in CRC, in which biallelic alterations are present in only approximately 3% of cases [89], whereas monoallelic mutations in HR genes are found in as many as 86% of cases, more commonly in *BRCA2*, *ARID1A*, ataxia-telangiectasia mutated (*ATM*) and BRCA1 associated RING domain 1 *(BARD1)* [90]. Another recent study revealed that among 9321 CRC patients, 1270 (13.6%) and 8051 (86.4%) were HRd and HR proficient, respectively, when a 33-gene panel was used [12]. Furthermore, when considering MMRp tumours, only 9.5% exhibited HRd characteristics, alongside a high tumour mutational burden (TMB), PD-L1 positivity and infiltration by immune cells and fibroblasts [12]. In the TRIBE2 trial, patients with MMRp/HRd tumours (10.7%) exhibited longer overall survival upon intensive treatment with the FOLFOXIRI triplet regimen than did patients with MMRp/HR-proficient tumours [12]. An additional analysis of patient data revealed that patients with MSS/HR-mutated tumours had significantly longer overall survival (OS) after ICB treatment than did those with MSS/HR-proficient tumours. The MSS/HR mutation group also presented a high TMB alongside infiltration of CD4 + T cells, which likely explains the anticancer immune response [91]. BRCA1- and BRCA2-altered tumours have shown enhanced immunosurveillance in several preclinical studies, but a correlation between ICB treatment and patient outcome was not evident [92]. In a pancancer cohort of patients treated with ICB from the Memorial Sloan Kettering Cancer Centre, BRCA1 was not associated with OS, whereas patients with BRCA2-altered tumours had a longer OS than those with wild-type BRCA2 [92]. Interestingly, patients with a low TMB but *BRCA2* mutation had the same OS as patients with highly mutated tumours did, suggesting neoantigen-independent mechanisms of immunogenicity [92]. Importantly, comutations in DDR pathways such as the HR and MMR pathways or the HR and base excision repair (BER) pathways are associated with increased TMB, neoantigen levels, and immune responses [93]. These findings were confirmed by analysing publicly available genomic data from a cohort treated with ICB [94]. These analyses revealed that DDR pathways were mutated in 36.4% of CRCs and that DDR mutations were associated with higher TMB levels and increased immune cell infiltration and immune checkpoint molecule expression [94]. Moreover, survival analysis revealed that DDR mutation was correlated with longer OS in patients with CRC treated with ICB. Overall, the impact of alterations in a single DDR pathway other than MMR on CRC immune surveillance remains largely undetermined.

### Can chemotherapy turn cold CRC (immune refractory) into hot CRC (immune responsive)?

The connection between DNA damage induced by chemotherapy and/or radiotherapy and immune surveillance is a focal point of multiple investigations. These studies are aimed to identify potential strategies to increase immunogenicity in immune-refractory tumours [95, 96]. Such approaches deserve attention because they intercept nonantigen-mediated (e.g., release of cytosolic DNA and c-GAS/STING activation) and antigen-dependent (e.g., adaptive impairment of DNA repair and neoantigen generation) mechanisms of immune sensitization by inducing sustained DNA damage or interfering with DNA repair proficiency (Fig. 1). In this setting, chemotherapy and/or radiotherapy are used as priming agents that precede or are given in combination with ICB [95, 96]. There are clinical studies that emphasize how the addition of ICB to anti-VEGF therapy and chemotherapy is promising in terms of OS (12 months) and progression-free survival (PFS) (6 months) in MMRp CRC patients [58]. The AtezoTRIBE (NCT03721653) is a multicentric, open-label, controlled, phase 2 study in which patients were randomized to receive first-line FOLFOXIRI and bevacizumab with or without atezolizumab (an anti-PD-L1 antibody). In this study, the median progression-free survival in the overall population was 13.1 months (80% CI 12.5–13.8) in the atezolizumab group and 11.5 months (80% CI 10.0–12.6) in the control group, with a significant hazard ratio (0.71, 80% CI 0.58–0.87, *p* = 0.015); however, no significant difference in OS was found [97, 98]. Interestingly, a post-hoc exploratory analysis identified high TMB and immunoscore as biomarkers associated with benefit for MMRp CRCs treated with atezolizumab [99]. Similarly, the results from the phase II CheckMate 9 × 8 trial (NCT03414983) investigating the addition of nivolumab (anti-PD-1) to FOLFOX and bevacizumab in first-line therapy failed to demonstrate a PFS benefit, although favourable trends in both the ORR and PFS rate at 18 months were observed in the nivolumab arm [100]. Additionally, studies with comparable designs, such as the NIVACOR trial (NCT4072198), are currently investigating similar hypotheses in the same patient population (Table 1) [101]. These clinical results warrant a better selection of patients with MMRp/MSS CRC who may benefit from the addition of ICB to first-line chemotherapy.

**Table 1:** Summary of clinical trials investigating chemo- and/or radiotherapy in combination with ICB treatment in unselected or MMRp mCRC patients

Chemo-immunotherapy trials in first-line setting				
Study name	Phase	Description	Status/Results^a^	Reference(s)
AtezoTRIBE	II	Randomized, open-label, treatment with Atezolizumab plus FOLFOXIRI/Bevacizumab vs FOLFOXIRI/Bevacizumab in the first-line treatment of patients with mCRC	Complete mPFS: 13.1 vs 11.5 months (p: 0.015); ORR: 59 vs 64%: mOS: 33 vs 27.2 months (p: 0.136)	NCT03721653 [97, 98]
CheckMate 9×8	II/III	Randomized, open-label, treatment with Nivolumab plus FOLFOX/Bevacizumab versus FOLFOX/Bevacizumab in the first-line treatment of patients with mCRC	Complete mPFS by BICR: 11.9 months in both arms (p:0.3); 18-months-PFS rate: 28% vs 9% ; ORR: 60% vs 46% ; mDoR: 12.9 vs 9.3 months	NCT03414983 [100]
HCRN GI14-186	II	Single arm, treatament with Pembrolizumab plus FOLFOX in previously untreated mCRC patients	Complete mPFS: 8.8 months; ORR: 56.7%	NCT02375672 [161]
MEDITREME	Ib/II	Single arm, treatment with FOLFOX plus Durvalumab and Tremelimumab in first-line treatment of RAS-mutated mCRC patients	Complete 3-month-PFS rate: 90.7%; ORR: 64.5%; mPFS: 8.2 months	NCT03202758 [162]
NIVACOR	II	Single arm, Nivolumab plus FOLFOXIRI/Bevacizumab in first-line treatment of patients with RAS/BRAF mutated mCRC	Ongoing	NCT04072198 [101]
POCHI	II	Single arm, Pembrolizumab plus Xelox/Bevacizumab as first-line treatment in MSS mCRC patients with resected primary tumours presenting a high immune infiltrate	Ongoing	NCT04262687
METIMMOX	II	NordicFLOX vs alternate treatment with 2 cycles of NordicFLOX followed by 2 cycles of Nivolumab in previously untreated patients with MMRp/MSS mCRC	Ongoing	NCT03388190
METIMMOX-2	II	Single arm, alterate treatment with 2 cycles of NordicFLOX and 2 cycles of Nivolumab in previously untreated patients with MMRp/MSS mCRC	Ongoing	NCT05504252
CIFOXRC	II	Single arm, treatment with cetuximab plus anti-PD1 plus FOLFOX in previously untreated metastatic or locally advanced RAS/BRAF wt right-sided colon cancer	Ongoing	NCT05468 177
XELOX +Bev +Tislelizumab for First-line Treatment of MSS/ pMMR RAS-mutated mCRC	II	Single arm, treatment with XELOX/Bevacizumab plus Tislelizumab in previously untreated MSS/MMRp RAS-mutated mCRC patients	Ongoing	NCT05970 302
FOBECAMS	II	Single arm, treatment with Camrelizumab plus FOLFIRI/Bevacizumab in previously untreated MSS mCRC patients	Ongoing	NCT06176885
CLIMB	II	Single arm, neoadjuvant treatment with Atezolizumab + plus FOLFOX/Bevacizumab in patients with colorectal liver metastases (CRLM)	Complete No results available	NCT03698461
**Chemo-immunotherapy trials in chemo-refractory setting**				
**Study name**	**Phase**	**Description**	Status/Results*	**Reference(s)**
Pembrolizumab, Capecitabine, and Bevacizumab in Treating Patients With Microsatellite Stable Colorectal Cancer	II	Single arm, treatment with Pembrolizumab plus Capecitabine/Bevacizumab in patients with non-resectable MSS mCRC who have not responded to previous fluoropyrimidine treatment	Complete ORR: 5%; mPFS: 4.3 months	NCT03396926 [163]
TAS-102 Plus Nivolumab in Patients With Microsatellite Stable Refractory Metastatic Colorectal Cancer	II	Single arm, treatment with Nivolumab plus TAS-102 in chemorefractory MSS mCRC patients	Complete ORR: 0%.; irRC mPFS: 2.2 months	NCT02860546 [164]
MAYA	II	Single arm, treatment with Temozolomide in combination with Nivolumab and Ipilimumab in MGMT-silenced MSS chemorefractory mCRCs patients who achieved disease control with 2 cycles of Temozolomide	Complete 8-month PFS rate: 36%; ORR: 45%; mPFS: 7 months; mOS: 18.4. months	NCT03832621 [103]
ARETHUSA	II	Single arm, priming treatment with Temozolomide in MGMT-silenced MSS chemorefractory mCRC patients until progression. At progression, treatment with Pembrolizumab in patients whose tumours show TMB elevation	Ongoing	NCT03519412 [104]
BACCI	II	Randomized, Double-Blind, Placebo-Controlled Study of Capecitabine Bevacizumab Plus Atezolizumab or Placebo in patients with chemo-refractory mCRC	Ongoing	NCT02873195
KEYNOTE-651	Ib	Open-label, multi-cohort, treatment with Pembrolizumab plus Binimetinib (MEKi) or Pembrolizumab plus chemotherapy with or without Binimetinib in previously treated mCRC patients	Ongoing	NCT03374254
Temozolomide, Cisplatin, and Nivolumab in People With Colorectal Cancer	II	Single arm combination of Temozolomide, Cisplatin, and Nivolumab in chemorefractory MMRp mCRC patients	Ongoing	NCT04457284
Pembrolizumab With Pemetrexed and Oxaliplatin in Chemo-Refractory Metastatic Colorectal Cancer Patients	Ib	Single arm, treatment with Pembrolizumab plus Pemetrexed and Oxaliplatin in MSS chemorefractory mCRC patients	Ongoing	NCT03626922
**Radio(Chemo)-immunotherapy trials**				
**Study name**	**Phase**	**Description**	Status/Results*	**Reference(s)**
Nivolumab+Ipilimumab+RT in MSS mCRC	II	Single arm combination of RT (3 doses during week 1) plus ipilimumab and nivolumab in chemorefractory MSS mCRC patients	Ongoing	NCT04575922
Combined Immunotherapy and Radiosurgery for Metastatic Colorectal Cancer	I	Single arm combination of RT (21 Gy in 3 doses) on liver metastases plus Ipilimumab and Nivolumab plus intratumoral and subcutaneous injection of CMP-001 (TLR9 agonist) in chemorefractory MSS mCRC patients	Ongoing	NCT03507699
SIRTCI	II	Single arm, treatment with Selective Internal Radiation Therapy (SIRT, TheraSphere®) plus Atezolizumab plus XELOX/Bevacizumab in mCRC patients with liver-dominant disease	Ongoing	NCT04659382
Pembrolizumab in Combination With Stereotactic Body Radiotherapy for Liver Metastatic Colorectal Cancer	Ib	Single arm, pre-operative treatment with Stereotactic Body Radiotherapy (SBRT) on liver metastases plus pembrolizumab in mCRC patient’s candidate to surgical resection of CRLMs	Ongoing	NCT02837263

*MSS* microsatellite instability, *mDoR* median duration of response, *mPFS* median Progression-free survival, *ORR* overall response rate, *OS* overall survival, *RT* radiotherapy, *BICR* Blinded Independent Central Review, *irRC* immune-related Response Criteria.

^a^In case a control arm is present, results are expressed as experimental arm (containing ICB) vs control arm. Last accessed: March 25, 2024.

Preclinical studies have used a different approach to turn cold immune refractory MMRp/MSS tumours into hot tumours, by hijacking mechanisms of resistance to alkylating agents to induce hypermutation and foster immune surveillance [17, 102]. This approach has been clinically investigated in two independent phase II trials (ARETHUSA and MAYA) [103, 104]. Both trials are based on the emergence of MMR genetic defects as a mechanism of resistance to treatment with the alkylating agent temozolomide (TMZ) in patients with MMRp CRCs. Specifically, TMZ treatment was performed in patients whose cancers displayed silencing of O6-methylguanine-DNA methyltransferase (MGMT), which is a DNA repair enzyme with a key role in chemoresistance to O6-alkylating agents such as TMZ [17]. Notably, TMZ induces alkylation of guanine at O6-methylguanine, which mismatches with thymine during DNA replication. In the case of functional MMR, these mismatches are repaired, initiating a futile cycle. Conversely, if MMR is not functional, these mismatches are fixed in DNA, affecting the mutational landscape and consequently the neoantigen repertoire of cancer cells [105]. In the ARETHUSA trial, a cohort of 30 MGMT-silenced, MMRp and RAS-mutant metastatic CRC patients received TMZ treatment as priming therapy; at the time of disease progression, patients advanced to the ICB phase with pembrolizumab only in the case of a TMB increase above 20 mutations per megabase [104]. In ARETHUSA, the TMB and mutational signatures analysed in tissue biopsy samples and circulating tumour DNA revealed the induction of alterations in MMR genes and tumour hypermutation. In 94% of the cases where TMZ mutational signature emerged, a p.T1219I *MSH6* variant was detected. Results from the initial analysis revealed that among the first six patients treated with pembrolizumab, four experienced disease stabilization [104]. In the MAYA trial, patients who achieved disease control after two cycles (8 weeks) of TMZ treatment were enroled to receive a combination of ipilimumab, nivolumab and TMZ, with no mandatory biomarker assessment. Notably, the ORR was 45% in 33 evaluable patients who received combined chemoimmunotherapy, with an 8-month PFS rate (the primary endpoint of the trial) of 36% [103]. The studies (completed and ongoing) that use chemotherapy to turn a cold tumour into hot are listed in Table 1.

### Enhancing tumour microenvironement dynamics with radiotherapy

The effects of radiotherapy on the immune system of cancer patients have been extensively described in numerous reports [18, 106–108]. Radiation induces cell death and the release of damage-associated molecular patterns (DAMPs), such as calreticulin, high mobility group box 1 (HMGB1), ATP, and cytokines and chemokines, which modulate the TME through the infiltration of DCs, macrophages, cytotoxic T cells and suppressive immune cells such as regulatory T cells and myeloid-derived suppressor cells (MDSCs) [106, 107, 109–111].

The primary effect of DAMPs is on DC priming and the activation of adaptive immunity (CD4+ and CD8 + T cells and NK activation). Specifically, HMGB1, along with calreticulin, is known to be pivotal for immunogenic cell death (ICD) in CRC treated with oxaliplatin by triggering Toll-like receptor 4 in the immune compartment [112]. ATP release, which acts as a ‘find me’ signal for immature macrophages other than DCs, facilitates the recruitment of myeloid cells to the TME, and ultimately culminates in the activation of CD8 + T cells and γδ T cells (Fig. 1, red panel) [113]. Moreover, additional proteins can contribute to ICD in CRC, as demonstrated by treatment with a novel topoisomerase inhibitor that induces the release of Annexin A1 (ANXA1), as well as HMGB1 and calreticulin (Fig. 1, red panel) [114]. Numerous clinical and preclinical investigations have explored the combination of radiotherapy and immunotherapy for treatment of CRCs [115].

Radiotherapy also affects DNA integrity by creating double- and single-strand breaks, inducing micronuclei formation and cGAS-STING activation through the rapid entrance of cGAS into micronuclei after the loss of membrane integrity [18]. Importantly, the main downstream effect of ICD is the activation of the IFN-I response. The role of IFN-I response in radiotherapy-induced immunity has been studied by Burnette and colleagues, who demonstrated that IFNα/β is required for tumour eradication following local radiotherapy as well as for the cross priming of tumour- infiltrating dendritic cells enabling CD8 + T cell activation [116].

In the context of DNA damage tumours may undergo autophagy, a self-degradative process that is key for balancing sources of energy in response to nutrient stress and has an impact on several metabolic and cancer-promoting pathways [117, 118]. Autophagy has implications for adaptive immunity, as neoantigens can be delivered to the major histocompatibility complex class II (MHC-II) compartment and presented to CD4 + T cells via autophagosomes (Fig. 1, red panel) [119]. The inhibition of autophagy results in impaired proliferation and the disrupted function of T cell receptor-stimulated CD8+ and CD4 + T cells, respectively (Fig. 1, red panel) [120]. However, in CRC cell lines, DSBs trigger a pro-survival autophagy signal upon treatment with ionizing radiations (IR) in an ATM- and p53-dependent manner [121]. In addition, another study revealed that, in MSS CRC, autophagy is a mechanism of immune evasion; the authors reported that inhibiting a core autophagy gene, ATG16L1, restored IFN-driven immune responsiveness (Fig. 1, red panel) [122]. Despite this evidence, in CRC, the pro- or anti-tumoural role of autophagy is not well defined and depends on AKT and mTOR activation [123]. On this basis, whether and to what extent irradiation-driven autophagy in CRC can perturb immune attractiveness must be fully elucidated at the molecular level.

The direct contribution of radiotherapy to the neoantigen landscape in cancer has been debated. Combining ICB and radiation upregulates tumour-associated antigen-MHC complexes, enhances antigen cross-presentation in the draining lymph node, and increases T cell infiltration into tumour [124]. Furthermore, the functional analysis of an NSCLC patient treated with radiotherapy and an anti-CTLA-4 agent revealed an expansion of CD8 + T cells specific for a neoantigen encoded in a gene upregulated by radiation [125]. Cancer cells targeted by IR can represent a source of new peptides that are presented on the tumour cell surface [126]. The clonality of mutations is emerging as a key factor of response across several cancer types [127, 128]. Despite this evidence, we cannot exclude the possibility that radiation-derived subclonal neoantigens might increase tumour heterogeneity, thereby distracting the immune system [128]. However, the ability of radiotherapy to induce the release of DAMPs, rewire the TME, and trigger a IFN-I response could affect the response to ICB in irradiated tumours against both neoantigens and preexisting tumour-associated antigens (Fig. 1, red panel). Furthermore, we have evidence in other cancer types that radiation-induced neoantigens are capable of triggering a CD8+ cytotoxic response and immune surveillance even at the subclonal level (Fig. 1, blue panel) [129]; however further studies are needed to better dissect the impact of radiation-induced neoantigens on the immune response towards radiated tumours.

Interestingly, the antigen-independent immunogenic mechanisms elicited by radiotherapy are not limited to the irradiated fields and may also sustain immune surveillance towards cancer lesions localized outside the irradiated sites—the so-called *abscopal effect* [130]. In a phase II single-arm study, 24 chemorefractory MMRp metastatic CRC patients received durvalumab (anti-PD-L1), tremelimumab (anti-CTLA-4), and radiotherapy. Only 2 patients achieved an objective response, characterized by increased CD8 + T cell activation and proliferation, an effector memory phenotype, and the reinvigoration of exhausted cells. Importantly, treatment led to the shrinkage of distant nonirradiated CRC tumours [131]. Nonirradiated tumour shrinkage was also observed in two other studies involving MMRp metastatic CRC patients, despite the phenomenon occurring in a limited number of patients (1/11 with an ORR of 9% in the first study [132] and 4/27 patients with an ORR of 15% in the second study [133]). Several studies are currently testing this hypothesis by combining hypofractionated radiotherapy at primary or metastatic disease sites with ICB, with or without chemotherapy (Table 1).

Although most of these approaches are still being investigated, attempts to increase the immunogenicity of MMRp CRC via chemotherapy/radiotherapy have not led to transformative results, suggesting that additional strategies should be pursued. For a list of completed and ongoing studies exploiting this treatment approach, see Table 1.

### Can PARP inhibition spark antitumour immunity in CRC?

In addition to chemotherapy and radiotherapy, the direct blockade of DDR pathways is being considered in CRC [134]. A clear cell autonomous effect of PARP inhibitors has been demonstrated in non-CRC tumours [135, 136]; however, recent findings suggest that the efficacy of PARP inhibitors may depend on STING-driven CD8 + T cell recruitment in triple-negative breast cancer [137]. Given that approximately 10% of MMRp CRCs harbour alterations in HR, it is imperative to address whether similar results can be achieved in CRC. Although PARP1 is the most studied isoform, 17 other isoforms that share homology with the catalytic domain of PARP1 have been identified [138]. PARP1 is generally activated during the early phase of DNA damage recognition to repair SSBs. Binding to altered DNA increases the catalytic activity of PARP1, which uses nicotinamide adenine dinucleotide (NAD + ) as a substrate to synthesize polymers of poly (ADP-ribose) transferred to PARP itself, histone H1, or other transcription factors [5]. The main mechanism of action of PARP inhibitors involves the impairment of PARP1 enzymatic activity and the “trapping” of inactive PARP1 on ssDNA, which is correlated with the cytotoxic potential of different inhibitors [139]. Whether and to what extent PARP inhibitors may sensitize tumour cells to chemotherapy was demonstrated by Wei and colleagues. They investigated the effect of Src homology-2 domain-containing protein tyrosine phosphatase-2 (SHP2) on tumour cell–intrinsic STING pathway activity and DNA repair in colon cancer. SHP2 was able to bind dephosphorylated PARP after DNA damage, preventing DNA repair and activating the STING pathway [140]. Importantly, the authors demonstrated in vitro and in vivo that an agonist of SHP2, lovastatin, led to excessive DNA damage and STING pathway activation enhancing the efficacy of irinotecan in preclinical CRC models. Sheng and colleagues demonstrated that PARP inhibition induced cytosolic double-stranded DNA, thereby activating the STING pathway and promoting tumour-infiltrating lymphocytes and antitumour immunity [141]. These antitumoural effects were further enhanced through ICB [141]. Recently, senescence has emerged as a cell-autonomous effect of PARP inhibitors in CRC [142]. Treatment with talazoparib (a PARP inhibitor) restricted p53 ubiquitination and activated p21-induced senescence in multiple CRC cell lines [142]. This phenotype was increased when a CDK4/6 inhibitor was used in combination with PARP inhibitor, resulting in the increased infiltration, activation, and proliferation of CD8 + T cells. Furthermore, the release of IFN-I cytokines (IFN-α and IFN-β) and IFN-γ, which regulate PD-L1 expression, led to the clearance of senescent cells after the addition of anti PD-L1, ultimately resulting in a potent antitumour effect [142]. Additionally, findings from murine preclinical models have shown that MEK inhibitors amplify DNA damage induced by PARP inhibitors, cytosolic DNA accumulation, STING activation and of CD8 + T cell recruitment [143]. Moreover, MEK inhibitors decrease the infiltration of MDSCs by reducing the release of IL-6 and GM-CSF [143]. Notably, the PARP inhibitor niraparib, combined with IR, produces excessive cytoplasmic double-stranded DNA in CRC, which is sensed by cGAS, thereby eliciting a cGAS-mediated antitumour immune response through the increased infiltration and activation of cytotoxic CD8 + T cells [144]. Caster and colleagues reported that pretreatment with veliparib (a PARP inhibitor) significantly augmented the increase in major histocompatibility complex class I (MHC-I) and PD-L1 expression in CT26 and MC38 tumours treated with IR. The concurrent administration of veliparib and radiation therapy substantially delayed tumour growth induced by anti-PD-1 therapy (Fig. 1, green panel) [145]. A case report described the efficacy of combined treatment involving a PARP inhibitor and immunotherapy in a patient with MSS mCRC carrying a *BRCA2* mutation, showing a major response to treatment with the PARP inhibitor olaparib and the anti-PD-1 agent tislelizumab [146]. The combination of ICB with PARP inhibitors in patients with HRd mCRC is currently being investigated in several clinical trials (Table 2). Interestingly, in the PEMBROLA trial (NCT05201612), the definition of HRd as a relevant biomarker was performed via a functional test of HR proficiency (the RAD51 score), which was previously shown to perform better than the genomic test in identifying PARP inhibitor sensitive breast cancers [147].

**Table 2:** Summary of clinical trials investigating DDR inhibitors in combination with ICB treatment in unselected or MMRp colon and rectal cancer patients. All treatment settings are included

Study name	Phase	Description	Status/Results	Reference(s)
PEMBROLA	II	Single arm, treatment with Olaparib and Pembrolizumab in HR Deficient [defined as the presence of a BRCA deleterious mutation and/or a low RAD51 score (cut-off <10%)] mCRC patients who have previously received treatment with oxaliplatin	Ongoing	NCT05201612
KEYLYNK-007	II	Single arm, treatment with Olaparib and Pembrolizumab in HRR mutated and/or HRD-positive advanced cancer (open to all solid tumours, including CRC)	Ongoing	NCT04123366
DAPPER	II	Randomized, open-label treatment with the combination of Durvalumab with either Olaparib or Cediranib (antiangiogenic TKI) in previously-treated MMRp mCRC patients (CRC cohort)	Ongoing	NCT03851614
Niraparib and Dostarlimab plus RT for rectal cancer	Ib/II	Single arm, neoadjuvant treatment with Niraparib and Dostarlimab plus hypofractionated RT for locally-advanced rectal cancer	Ongoing	NCT04926324
Veliparib, Pembrolizumab and Chemotherapy in Locally Advanced Rectal Cancer	II	Single arm, neoadjuvant treatment with Veliparib and Pembrolizumab plus XELOX or FOLFOX for locally-advanced rectal cancer	Ongoing	NCT02921256

Last accessed: March 25, 2024.

*MMRp* mismatch repairproficient, *mCRC* metastatic colorectal cancer, *TKI* tyrosine-kinase inhibitor.

Overall, targeting synthetic lethal vulnerabilities in HRd CRCs via PARP inhibition with or without other DNA damage treatments (Table 2) is emerging as a promising strategy to increase immunogenicity in otherwise immune refractory MMRp CRCs.

### Combining standard-of-care therapeutic options with DDR targeting to enhance the immune recognition of CRCs

Current therapeutic options for mCRC patients are based on various chemotherapeutic agents such as 5-fluorouracil (5-FU)/capecitabine, irinotecan, and oxaliplatin, which can be combined with targeted agents such as bevacizumab, cetuximab, or panitumumab [148]. Among these drugs, oxaliplatin causes interstrand and intrastrand DNA cross-links that block DNA replication and transcription, leading to apoptotic cell death and consequently to ICD [112]. Interestingly, a loss-of-function genetic screening of kinomes revealed that Ataxia telangiectasia and Rad3-related protein (ATR) inhibition synergizes with oxaliplatin to induce cancer cell death [149]. In addition, the authors highlighted the synergistic effects of oxaliplatin and the ATR inhibitor VE-822 in many colorectal cancer cell lines, demonstrating the occurrence of cytosolic DNA release, CD8 + T cell infiltration and reduced tumour growth in immune competent mice [149]. While oxaliplatin can induce ICD and the biological features typical of this peculiar type of tumour cell death, irinotecan-mediated killing does not occur via ICD [150, 151]. However, although the cytotoxic effect of irinotecan is known to be linked to nonimmunogenic mechanisms, recent evidence indicates that treatment with SN38 (the active metabolite of irinotecan) impacts MHC-I exposure and the expression of NK ligands [152].

ATR plays a central role in the cellular replication stress response by activating cell-cycle checkpoints and DNA replication processes to control cell division and safeguard genomic integrity. ATR is activated in the presence of single-stranded DNA (ssDNA) breaks at sites of stalled replication forks or in resected double strand DNA ends inducing a cascade of events that lead to cell cycle arrest and replication fork stabilization, thereby permitting DNA repair [153]. The rationale behind targeting ATR in cancer is that the inhibition of ATR may lead to genomic instability, DSBs, and replication fork collapse. Novel findings suggest that the ATR inhibitor ceralasertib (AZD6738) induces micronuclei formation and radiosensitization in preclinical models [154]. In another study, the same ATR kinase inhibitor combined with radiation therapy attenuated radiation-induced CD8 + T cell exhaustion and potentiated CD8 + T cell activity in CT26. Moreover, ceralasertib blocked radiation-induced upregulation of PD-L1 expression on tumour cells and dramatically decreased the number of tumour-infiltrating regulatory T cells [155]. The relevance of inhibiting ATR was also demonstrated in a study in which WEE1 was also targeted [156]. WEE1 is a protein kinase that regulates the G2/M cell cycle checkpoint, causing cell cycle arrest so that DNA damage repair can be properly executed [157]. The authors reported that the dual inhibition of ATR and WEE1 promoted cytosolic double-strand DNA, which activated the STING pathway and led to the production of IFN-1, thereby favouring CD8 + T cell infiltration [156]. Finally, in that study, the dual inhibition of WEE1 and ATR induced PD-L1 expression on tumour cells, and blocking PD-L1 enhanced the effects of chemotherapies [156].

Another potential target is ATM, which is activated during radiation and promotes DNA damage repair through the HR and NHEJ pathways in a cell cycle-dependent manner [158]. However, despite evidence supporting the cell-autonomous effect of ATM inhibition on radiosensitization, its impact on the immune microenvironment remains largely unclear [159].

Overall, the combination of cytotoxic agents and/or radiotherapy with DDR inhibitors such as ATR inhibitors is emerging as a potential new avenue to leverage antigen-independent immunogenic mechanisms with the aim of enhancing anticancer immune surveillance.

## Conclusions

Currently, the vast majority (95%) of mCRC patients are not eligible for immunotherapy. Accordingly, increasing the fraction of patients who may benefit from the transformative curative potential of immunotherapy constitutes an unmet and urgent clinical need in the CRC field. Emerging evidence highlights how DNA repair alterations, beyond MMR, can create a permissive microenvironment by (a) triggering intracellular pathways that developed to restrict pathogen infection and are capable of unleashing the immune system against cancer cells; (b) inducing immunogenic cell death or autophagy in cancer cells; and (c) modulating immune checkpoint molecules, thereby allowing ICB engagement. This paves the way for exploring whether the presence of altered DNA repair pathways, other than MMR, may be promising molecular substrates for enroling or stratifying patients in immune-activating therapeutic programmes. Inhibiting DDR mechanisms in cancer is considered a promising strategy and many clinical trials are currently testing the efficacy of these drugs in establishing immune sensitization [160]. Overall, chemotherapy and radiotherapy remain the primary therapeutic options for mCRC patients. However, evidence that a better characterization of the DDR pathways beyond MMR may be relevant to inform a more rational therapeutic approach for CRC is accumulatimg both at the preclinical and clinical levels. In the next few years, identification of CRCs in which the occurrence of dysfunctional DDR pathways leads to immune surveillance could increase the fraction of patients benefitting from immunomodulatory therapies.
